# Editorial: Safety and security of robotic systems: intelligent algorithms

**DOI:** 10.3389/fnbot.2023.1342742

**Published:** 2024-01-08

**Authors:** Chengwei Wu

**Affiliations:** Department of Control Science and Engineering, Harbin Institute of Technology, Harbin, China

**Keywords:** safety, security, robotic systems, intelligent algorithms, cyber attacks

Robots have found widespread applications across diverse fields, including military, smart manufacturing, transportation, healthcare, and smart homes. The increasing ubiquity of intelligent devices and communication networks, however, presents certain challenges for the control of robot systems. First, modern robotic systems can be conceptualized as intricate information-physical systems designed with goals that are not only vast but also varied rather than singular. The potential for conflicts among these objectives underscores the importance of thoughtfully designing approaches to achieve extensive and diverse safety and performance goals. Second, insights drawn from various network attack cases, such as Stuxnet and RQ-170 attacks, highlight the critical significance of developing robust security solutions for contemporary robotic systems.

In the present day, research on this Research Topic is garnering increased attention and has produced various noteworthy achievements, which have been primarily reported with the assistance of precise system knowledge. The advent of the artificial intelligence era has proven advantageous in addressing safety and security concerns in robotic systems through the implementation of learning-based intelligent algorithms.

The Research Topic aims to provide researchers with a forum for gathering the latest research outcomes in the area of safety and security in robotic systems. This Research Topic of four high-quality papers showcases the most recent advancements in the field of control of robotic systems.

The paper titled “*Face morphing attack detection based on high-frequency features and progressive enhancement learning*” by Jia et al. describes a detection method that leverages high-frequency features and progressive enhancement learning. This technique extracts high-frequency information from the three-color channels of an image to precisely capture details and texture changes. Simultaneously, a progressive enhancement learning framework is devised that integrates self-enhancement and interactive enhancement modules. These modules systematically amplify features to capture subtle deformation traces by seamlessly merging high-frequency information with RGB details. Experimental results on standard databases, in comparison with nine classical techniques, showcase the exceptional performance of the proposed method.

“*Research on control strategy of vehicle stability based on dynamic stable region regression analysis*” by Liu et al. presents a support vector regression (SVR) model for the automated regression of the dynamic stable region and devises a direct yaw-moment control (DYC) stability controller based on linear time-varying model predictive control (LTV-MPC). The impact of critical factors, such as the center of mass position and road adhesion coefficient, on the stable region was investigated through phase diagrams. Simulation experiments substantiate the effectiveness of the stability assessment and control algorithm.

The work presented by Yue et al., “*Research on reinforcement learning-based safe decision-making methodology for multiple unmanned aerial vehicles*,” reports on a Transfer Safety Soft Actor-Critic (TSSAC) for decision-making in multiple UAVs. The decision-making process for each drone was modeled using a constrained Markov decision process (CMDP), where safety constraints were imposed to maximize returns. Within the CMDP model, the soft actor-critic-Lagrangian (SAC-Lagrangian) algorithm was integrated with an enhanced Lagrangian multiplier. Furthermore, parameter-based transfer learning was applied to facilitate collaborative and efficient training for multiple UAV missions. Simulation results demonstrate that the proposed method enhances the safety and training efficiency of UAVs, enabling adaptability to dynamic scenarios.

Finally, in the work “*Efficacy and safety study of wearable cyborg HAL (hybrid assistive limb) in hemiplegic patients with acute stroke (EARLY GAIT study): protocols for a randomized controlled trial*,” Watanabe et al. propose a randomized controlled trial aimed at clarifying the effectiveness and safety of gait therapy in acute stroke hemiplegic patients using the wearable cyborg Hybrid Assistive Limb (HAL) compared to conventional gait training (CGT). Additionally, the study aimed to formulate clinical trial protocols for acute stroke initiated by physicians based on the research findings. If the results demonstrate the effectiveness of the proposed treatment on the prognosis of hemiplegic acute stroke patients, this study could pave the way for the adoption of the HAL system as an effective tool in future stroke rehabilitation programs for these patients.

This Research Topic reports on various research endeavors in the field of robotic system safety and security. Robotic systems control research methods demonstrate significant potential for addressing practical challenges. With ongoing innovations and improvements in research methods and algorithms, learning-based technologies are poised to contribute critical theories and provide new results to address key aspects of this topic and solve experimental problems in the future.

## Author contributions

CW: Writing – review & editing.

